# Injection of Lidocaine With Epinephrine for Bee Sting Large Local Reactions

**DOI:** 10.1016/j.acepjo.2024.100009

**Published:** 2025-01-08

**Authors:** Keith A. Denkler, Rosalind F. Hudson

**Affiliations:** 1 Division of Plastic Surgery, University of California, San Francisco, California, USA; 2 Private Practice Internal Medicine, Larkspur, California, USA

**Keywords:** Hymenoptera sting, Hymenoptera large local reaction, localized allergy, epinephrine Hymenoptera sting

## Abstract

Bee stings are very common worldwide. About 5% to 15% of those afflicted have a large local reaction, defined as a skin reaction around the sting site with edema, erythema, itching, and an injury diameter >10 cm. Standard treatments for large local reactions include ice, nonsteroidal anti-inflammatory medications, antihistamine medications, and topical or systemic corticosteroids, none of which immediately treat the pain associated with the symptoms nor initiate immediate treatment of the allergic and inflammatory response. We present a dramatic and rapid reversal of a periorbital large local reaction treated with subcutaneous and intradermal injection of 1% lidocaine with epinephrine into the sting area. The lidocaine rapidly reversed the symptoms of pain and pressure, and the low dose of epinephrine, within 2 hours, significantly reversed the allergic periorbital and eyelid edema. No further symptoms evolved, suggesting that the epinephrine terminated the allergic cascade.

## 1 Introduction

Large local reactions (LLR) after bee stings are common worldwide and occur in 5% to 15% patients.^1^^,^^2^ The prominent inflammatory response is caused by the venom’s neuropeptides, histamine, and proinflammatory compounds and can take 5 to 10 days to resolve.^1^^,^^2^ Standard treatments for LLR include ice, nonsteroidal anti-inflammatory medications, antihistamine medications, and topical or systemic corticosteroids, none of which immediately treat the pain associated with the symptoms nor initiate immediate treatment of the allergic and inflammatory response.^1^^,^^2^

The risk of anaphylaxis in patients who had LLR to Hymenoptera stings has been studied in a retrospective and prospective study of 477 patients.^3^ The investigators found a correlation between the number of LLRs and the risk of developing anaphylaxis. In patients who had a single LLR as the first manifestation of venom allergy, there was a risk, albeit low, of a serious reaction to a subsequent Hymenoptera sting, whereas there was no risk of anaphylaxis in patients who had a history of 2 previous consecutive LLRs.

The past medical history of patients who presented with LLR after Hymenoptera stings should include specific inquiry into the risk factors for anaphylaxis: a history of anaphylaxis, mast cell disease, or asthma (coexisting or uncontrolled asthma). If any of these past medical history risk factors are obtained during the evaluation and treatment of a patient who has an LLR, an adrenaline auto-injector prescription is warranted as well as a referral to an Allergist/Immunologist is indicated for consideration of allergen immunotherapy.^4^^,^^5^

## 2 Case

**Figure 1:**
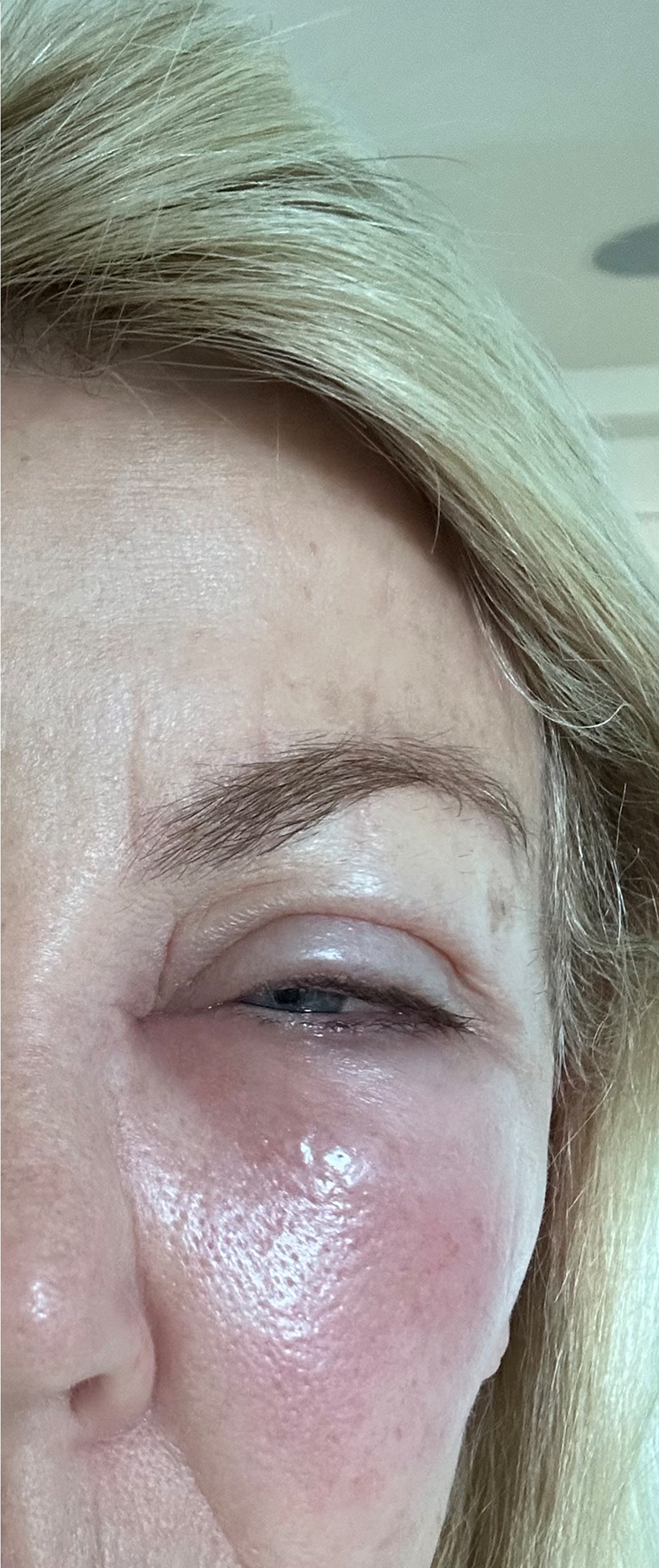
Twenty hours after a bee sting to the lower left orbital/cheek area.

**Figure 2:**
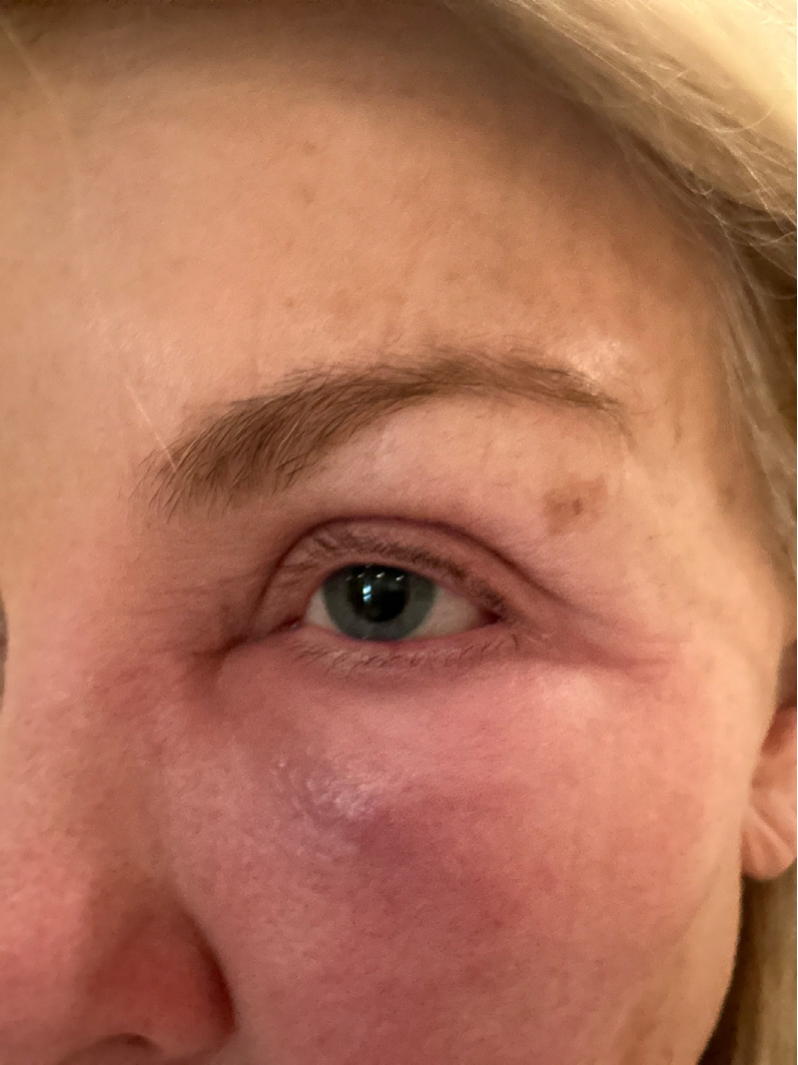
Two hours after injection of 1.5 cc of 1% lidocaine with epinephrine into the area of the orbital/cheek bee sting.

A recreational beekeeper presented with a left periorbital LLR 20 hours after a bee sting. The patient had a history of multiple LLRs in the past. She complained of worsening left periorbital pain, pressure, redness, and swelling of the upper and lower eyelids to the point that she could no longer open her left eye (Fig 1). There were no signs or symptoms suggesting a risk of anaphylaxis. As treatment, the lower orbital puncture area of the sting was infiltrated with 1.5 mL 1% lidocaine with epinephrine 1: 100,000 using 1 mL subcutaneously and half mL intradermally. Within minutes of the lidocaine with epinephrine injection, the patient stated multiple times, “I feel 100% better,” as pain and pressure were completely relieved. Two hours after injection, the patient’s periorbital edema had dramatically resolved, and upper eyelid edema had resolved even though the injection was into the lower orbital area (Fig 2). There was no allergic rebound after the epinephrine effects had resolved that evening. The next morning, the patient was asymptomatic except for mild lower eyelid skin edema. The progression of the patient’s allergic and inflammatory cascade appeared to have ceased.

## 3 Discussion

Epinephrine treatment of anaphylaxis is well described and lifesaving for bee sting anaphylaxis, but use of local anesthetic with epinephrine in local anesthetic form for LLR is mentioned only once in 1972 and reiterated in 1990 as a treatment for the pain of Hymenoptera sting local reactions.^6^^,^^7^ Additionally, our literature review could not find any mention of the effects of localized injection of low-dose epinephrine (with or without lidocaine) on the allergic cascade.

The allergic cascade is initiated when the allergen binds to antigen-specific immunoglobulin E on the surface of mast cells, causing immediate histamine release. Epinephrine is the only drug that can stabilize mast cells, prevent histamine degranulation, and indirectly diminish the histamine effect on target cells.

In this patient who had a left periorbital LLR, treatment with subcutaneous and intradermal infiltration of 1% lidocaine with epinephrine 1:100,000 into the sting area immediately relieved pain and pressure and rapidly decreased the allergic and inflammatory periorbital and eyelid edema. We suspect that the rapid improvement in this patient demonstrates that the epinephrine injection worked rapidly to prevent histamine degranulation, counteract the effects of histamine on target cells, and create local vasoconstriction.

We hypothesize that epinephrine is scalable. This patient demonstrates that a small dose of epinephrine 1:100,000 can treat a large local reaction*,* just as a large dose of epinephrine 1:1,000 can treat a systemic reaction. The question of which patients who had LLRs to Hymenoptera stings will go on to have systemic reactions was studied by Pucci et al^3^ in 2015. Of 477 patients, 396 had a history of systemic reactions, with 95.8% presenting with systemic reactions as their first manifestation of allergy and 4.2% presented with a single LLR as their first manifestation of allergy but subsequently went on to have systemic reactions. In the LLR group, 81 patients presented only with LLRs and had previously had at least 2 LLRs. The authors concluded that patients who had repeated LLRs to stings had no risk of systemic reactions, whereas patients who had a single LLR were not excluded from such risks. Our case report presents a patient who had a history of multiple LLRs and no history of anaphylaxis, making her risk of anaphylaxis very low.

The past medical history of patients who presented with LLR should include specific inquiry into the risk factors for anaphylaxis: a history of anaphylaxis, mast cell disease, or asthma (coexisting or uncontrolled asthma). If any of these past medical history risk factors are obtained during the evaluation and treatment of a patient who has an LLR, an adrenaline auto-injector prescription is warranted as well as referral to an Allergist/Immunologist is indicated for consideration of allergen immunotherapy.^4^^,^^5^

Our observational experience demonstrates the efficacy of 1% lidocaine with epinephrine for treating a periorbital LLR caused by a bee sting. Further research is needed to investigate the treatment of acute LLR with local injection of lidocaine combined with epinephrine and confirm if this consistently terminates pain and enables rapid resolution of allergic symptoms in other bites and stings.

## Funding and Support

By *JACEP Open* policy, all authors are required to disclose any and all commercial, financial, and other relationships in any way related to the subject of this article as per ICMJE conflict of interest guidelines (see www.icmje.org). The authors have stated that UCSF provided payment for open access in accordance with faculty open access paper submissions.

## Conflict of Interest

All authors have affirmed they have no conflicts of interest to declare.
